# Genomic Comparison, Phylogeny and Taxonomic Reevaluation of the *Ectothiorhodospiraceae* and Description of *Halorhodospiraceae* fam. nov. and *Halochlorospira* gen. nov.

**DOI:** 10.3390/microorganisms10020295

**Published:** 2022-01-26

**Authors:** Johannes F. Imhoff, John A. Kyndt, Terrance E. Meyer

**Affiliations:** 1 GEOMAR Helmholtz Centre for Ocean Research Kiel, Düsternbrooker Weg 20, 24105 Kiel, Germany; 2 College of Science and Technology, Bellevue University, Bellevue, NE 68005, USA; jkyndt@bellevue.edu; 3 Department of Biochemistry, University of Arizona, Tucson, AZ 85721, USA; temeyer@email.arizona.edu

**Keywords:** purple sulfur bacteria, genomic phylogeny, *Ectothiorhodospiraceae*, *Halorhodospiraceae*, new family and genus

## Abstract

The *Ectothiorhodospiraceae* family represents purple sulfur bacteria of the *Gammaproteobacteria* found primarily in alkaline soda lakes of moderate to extremely high salinity. The main microscopically visible characteristic separating them from the *Chromatiaceae* is the excretion of the intermediate elemental sulfur formed during oxidation of sulfide prior to complete oxidation to sulfate rather than storing it in the periplasm. We present a comparative study of 38 genomes of all species of phototrophic *Ectothiorhodospiraceae*. We also include a comparison with those chemotrophic bacteria that have been assigned to the family previously and critically reevaluate this assignment. The data demonstrate the separation of *Halorhodospira* species in a major phylogenetic branch distant from other *Ectothiorhodospiraceae* and support their separation into a new family, for which the name *Halorhodospiraceae* fam. nov. is proposed. In addition, the green-colored, bacteriochlorophyll-containing species *Halorhodospira halochloris* and *Halorhodospira abdelmalekii* were transferred to the new genus *Halochlorospira* gen. nov. of this family. The data also enable classification of several so far unclassified isolates and support the separation of *Ectothiorhodospira shaposhnikovii* and *Ect. vacuolata* as well as *Ect. mobilis* and *Ect. marismortui* as distinct species.

## 1. Introduction

The phototrophic purple sulfur bacteria are *Gammaproteobacteria* that use sulfide and other reduced sulfur sources as photosynthetic electron donors and oxidize these to sulfate as the final oxidation product. The genus *Ectothiorhodospira* originally was included in the *Chromatiaceae* family [1]. Phenotypic differences and the distinction of *Ectothiorhodospira* from *Chromatiaceae* by oligonucleotide patterns of 16S rRNA molecules [2,3] led to their separation in a distinct family, the *Ectothiorhodospiraceae* [4].

The two families of purple sulfur bacteria can be distinguished by the most obvious differences in the oxidation of sulfide which are microscopically visible, the formation of elemental sulfur globules inside the cells in *Chromatiaceae* and outside the cells in *Ectothiorhodospiracae* [5,6,7,8]. Other characteristic properties that distinguish species of both families are different internal membrane systems, which are in the form of vesicles in *Chromatiaceae* and in the form of membrane stacks in *Ectothiorhodospiraceae* [5,6,7], and by a number of chemotaxonomic properties, including the quinone, lipid and fatty acid composition [9,10] and lipopolysaccharide structures [11,12,13]. Sequence analysis of the 16S rRNA gene from available type strains of *Ectothiorhodospiraceae* had demonstrated the clear divergence of two groups within this family which had been recognized as two different genera: slightly to moderately halophilic species of *Ectothiorhodospira* and extremely halophilic species, which were transferred to the new genus *Halorhodospira* [14].

Further comprehensive phylogenetic studies, including large numbers of phototrophic purple bacteria of *Alpha*-, *Beta*- and *Gammaproteobacteria*, comparing sequences of 16S rRNA genes and *bchXYZ* genes [15], *pufLM* genes [16] and the glycine/sarcosine methyltransferase essential for glycine betaine biosynthesis [17] all supported the recognition of three major groups of purple sulfur bacteria, the *Chromatiaceae*, the *Ectothiorhodospiraceae* (except *Halorhodospira* species) and the extremely halophilic *Halorhodospira* species.

In this communication we compare the genome information of a large number of available strains and species of the *Ectothiorhodospiraceae*, highlight some properties related to sulfur metabolism, electron transport and photosynthesis and discuss the phylogenetic and taxonomic status of these bacteria. The data are in support of a distinction of the *Halorhodospira* species from *Ectothiorhodospiraceae* and *Chromatiaceae* on a family level. The name *Halorhodospiraceae*, fam. nov. is proposed for this new family. *Ectothiorhodospiraceae* and *Halorhodospiraceae* families are defined. In addition, the separation of *Halo-rhodospira halochloris* and *Halorhodospira abdelmalekii* into the new genus *Halochlorospira* gen. nov. as *Halochlorospira halochloris* and *Halochlorospira abdelmalekii* is proposed.

According to genome information, including ANI, most of the chemotrophic genera, which have been assigned in recent years in the literature and in databases to the *Ectothio-rhodospiraceae,* should be excluded from this family due to their large phylogenetic distance and significantly different phenotypic properties.

## 2. Material and Methods

### 2.1. Genome Sequences Used in this Study

Several genome sequences from phototrophic *Ectothiorhodospiraceae* and *Halo-rhodospiraceae* species have been published before [17,18,19,20,21,22,23] and a number of additional genome sequences have been established during this study (Table 1). The genome sequences from other strains of phototrophic bacteria used in this study are the following: *Allochromatium humboldtianum* DSM 21881 (JABZEO000000000), *Allochromatium vinosum* DSM 180 (CP001896), *Chromatium okenii* DSM 169 (NRRQ01000000), *Chromatium okenii* LaCa (PPGH01000000), *Halochromatium glycolicum* DSM 11080 (NRSJ01000000), *Halochromatium salexigens* DSM 4395 (NHSF01000000), *Marichromatium bheemlicum* DSM 18632 (JAAXKX000000000), and *Marichromatium gracile* DSM 203 (SMDC01000000). Those from chemotrophic species are: *Acidiferrobacter thiooxydans* m-1 (PSYR01000000), *Alkalilimnicola ehrlichii* MLHE-1 (CP000453), *Alkalispirillum mobile* DSM 12769 (RCDA01000000), *Aquisalimonas asiatica* CGMCC 1.6291 (FOEG01000000), *Arhodomonas aquaeolei* DSM 8974 (ARGF00000000), *Halofilum ochraceum* XJ16 (LVEG02000000), *Halopeptonella vilamensis* DSM 21056 (VMKO01000000), *Inmirania thermothiophila* DSM 100275 (RJVI01000000), *Nitrococcus mobilis* Nb-231 (AAOF00000000), *Oceanococcus atlanticus* 22II-S10r2 (AQQV00000000), *Spiribacter salinus* M19-40 (CP005963), *Thioalbus denitrificans* DSM 26407 (QPJY01000000), *Thioalkalivibrio sulfidophilus* HL-EbGr7 (CP001339), *Thioalkalivibrio versutus* AL2 (MVAR00000000), *Acidihalobacter prosperus* DSM 5130 (JQSG00000000), *Thiogranum longum* DSM 19610 (SMFX01000000), *Thiohalomonas denitrificans* HLD2 (FMWD01000000) and *Thiohalospira halophila* DSM 15071 (FOMJ01000000).

### 2.2. Genomic DNA Extraction and Sequencing

For the strains sequenced in this study, genomic DNA was prepared from frozen cells, using the GeneJET DNA purification kit (Thermo Fisher Scientific, Waltham, MA, USA). The quantity and purity of DNA were determined using Qubit and NanoDrop instruments and showed absorbance 260/280 ratios between 1.67 and 2.12. The DNA libraries were prepared with the Nextera DNA flex library prep kit (Illumina, Inc., San Diego, CA, USA). All genomes were sequenced using 500 μL of a 1.8pM library with an Illumina MiniSeq instrument, using paired-end sequencing (2 × 150 bp). Quality control of the reads was performed using FASTQC in BaseSpace (Illumina, version 1.0.0), using a kmer size of 5 and contamination filtering. The data for each was assembled de novo using Unicycler [24] in PATRIC [25]. The genome sequences were annotated using RAST (Rapid Annotations using Subsystem Technology; version 2.0; [26]).

### 2.3. Whole Genome Comparison

Average percentage nucleotide identity (ANIb) between the whole genomes was calculated using JSpecies [27]. JSpecies uses a pairwise genome comparison algorithm to measure the probability of genomes belonging to the same species, with an arbitrary species cutoff of 95%. The whole genome-based phylogenetic tree was generated within PATRIC [25], using the CodonTree pipeline which uses PGFams as homology groups. Among these selected genomes, 141 PGFams were found using the CodonTree analysis, and the aligned proteins and coding DNA from single-copy genes were used for RAxML analysis [28,29]. 100 rounds of bootstrapping were performed using the ‘Rapid bootstrapping option. The resulting Newick file was used in iTOL for tree visualization [30]. Average amino acid identity (AAI) values were calculated from the proteome comparison in PATRIC. Only bi-directional hits were used for this analysis and *Hlr. halophila* SL1^T^ *and Hlr. halochloris* DSM1059^T^ (BN9850) as the reference strains. Pairwise 16S rRNA comparisons were performed using LALIGN (EMBL-EBI), using the genome-derived 16S rRNA sequences.

The alignment for the 16S rRNA comparisons was performed using Clustal Omega [31] which uses seeded guide trees and Hidden Markov Model (HMM) profiles to generate multiple sequence alignments. The phylogenetic tree was calculated by the neighbour-joining (NJ) method [32] in JALVIEW [33] and a Newick file was generated. The Jalview NJ method uses the BLOSUM62 substitution matrix to compute a sum of scores for the residue (base) pairs at each aligned position. iTOL was used to draw the phylogenetic trees expressed in the Newick phylogenetic tree format [30]. Due to the lack of complete 16S rRNA sequences from whole genomes, Genbank 16S rRNA sequences were used instead for *Ect. salini* (FM244738.1), *Ect. imhoffii*, *Arhodomonas aquaeoli* (M26631.2) and *Nitrococcus mobilis* (NR_104912.1), for the 16S rRNA phylogenetic tree.

For the synteny analysis, comparative genome regions were generated using global PATRIC PGFam families to determine a set of genes that match a focus gene. The gene set is compared to the focus gene using BLAST and sorted by BLAST scores within PATRIC [25]. The Compare Region Viewer in PATRIC displays the focus gene along with the other genes in the same family and their flanking regions in their genomes. The *bch*B gene was used as a focus gene to analyze synteny of the photosynthetic and bacteriochlorophyll gene cluster.

## 3. Results and Discussion

With the availability of large numbers of genome sequences, the average nucleotide identity (ANI) of genomes became an established measure to compare the similarity/relatedness of bacteria and is an accepted alternative to the classical DNA–DNA hybridization method, which only provided reliable results if performed by experts familiar with this method. It has been suggested to recognize bacteria with ANI >95% as belonging to the same species [27], while bacteria with ANI <90% would be recognized in most cases as separate species. Those with values between 90 and 95% identity may be argued either way depending on other properties. As ANI is apparently more precise in the differentiation of closely related bacteria compared to the 16S rRNA gene sequences [27], we have analyzed ANI from all phototrophic *Ectothiorhodospiraceae* species with available genome sequences with representatives of the genera *Ectothiorhodospira*, *Ectothiorhodosinus*, *Thio-rhodospira* and *Halorhodospira.* In addition, representative chemotrophic *Ectothiorhodospiraceae* with available genome sequences were included.

As the species of *Halorhodospira* are clearly distinct from other *Ectothiorhodospiraceae* genera and the separation of *Halorhodospira* species on a family level is proposed, in the following, the term *Ectothiorhodospiraceae* will be used in the strict sense with the exclusion of *Halorhodospira* species and species of *Halorhodospira* will be treated separately.

### 3.1. Genomes of Ectothiorhodospiraceae

The *Ectothiorhodospiraceae* (excluding *Halorhodospira*) currently contain 10 named phototrophic species. The genome sequences of *Trs. sibirica* ATCC 700588^T^ [18], *Ect.* strain PHS-1 [19], *Ect. haloalkaliphila* ATCC 51935^T^ [20] and *Ect*. strain BSL-9 [21] have been previously published. In addition, genome sequence information for *Ect. magna* DSM 22250^T^, *Ect. marina* DSM 241^T^*, Ect. marismortui* DSM 4180^T^, *Ect. mobilis* DSM 237^T^*, Ect. shaposhnikovii* DSM 243^T^ and *Ectothiorhodosinus mongolicus* DSM 15479^T^ was presented in earlier publications [15,16,17]. Here we include additional genome sequences of type strains of *Ect. vacuolata* DSM 2111^T^ and *Ect. variabilis* DSM 21381^T^ and additional isolates (DG9, A-7R, A-7Y, B14B, WN21Y, WN21R, WN2R, BN9902 = C, BN9100 = YC6.1 and BN9905 = ScotB) and use this information for a comparison of species and strains (Table 1). Based on comparison of genome length and its G + C content (mol%), several groups of strains/species can be clearly distinguished (Table 1). The group containing *Ect. mobilis* DSM 237 and both *Ect. marismortui* strains (DSM 4180 and DG9) has a smaller genome size (2.62–2.80 Mbp) and higher percentage of 68.2–68.4 mol% G + C as compared to the majority of the genomes that have genome sizes from 3.20 Mbp to 3.79 Mbp (with the exception of *Ect*. spec. strain PHS-1 with 2.94 Mbp) and a G + C content of 62.3 to 63.7 mol%. *Ectothiorhodosinus mongolicus*, *Thiorhodospira sibirica* and *Ect. magna* apparently are more distinct and also have a lower G + C content (Table 1).

The ANI comparison of the phototrophic *Ectothiorhodospiraceae* (Table 2) clearly identifies three groups of species, with ANI values >80%: (i) including the type strains of *Ect. vacuolata*, *Ect. shaposhnikovii* and *Ect. magna* (colored orange in Table 2), with *Ect. magna* being clearly more distant to the others with ANI values between 83–87%; (ii) including the type strains of *Ect. haloalkaliphila*, *Ect. variabilis* and *Ect. marina* (colored yellow in Table 2); (iii) including the type strains of *Ect. mobilis* and *Ect. marismortui* (colored green in Table 2). More distant to these groups are *Ectothiorhodosinus mongolicus* and *Thiorhodospira sibirica*, with ANI values of <70% relative to the others.

**Figure 1:**
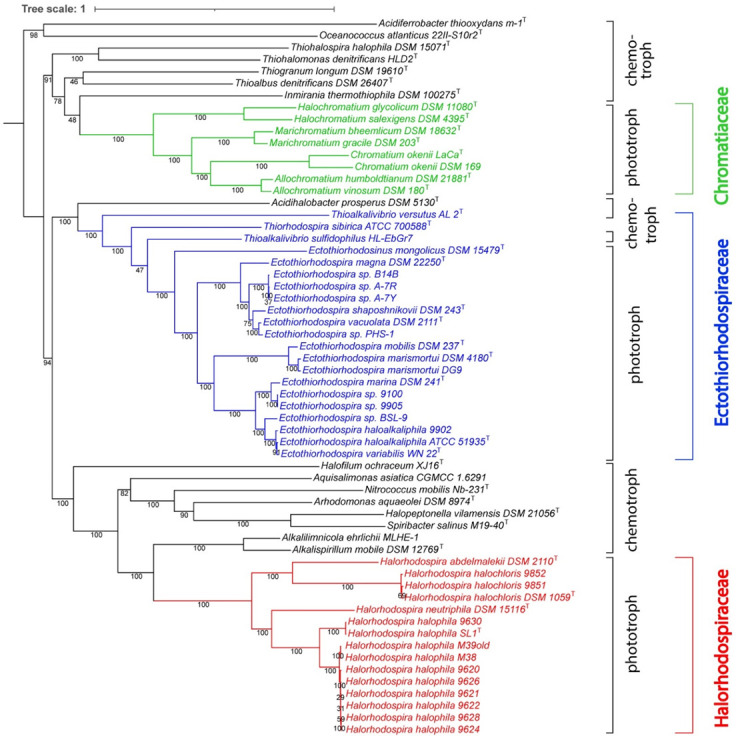
Whole genome-based phylogenetic tree of the *Ectothiorhodospiraceae*. The support values for the phylogenetic tree are generated using 100 rounds of the ‘Rapid bootstrapping’ option of RaxML. The tree was rooted at midpoint and the branch length tree scale is defined as the mean number of substitutions per site, which is an average across both nucleotide and amino acid changes. *Oceanococcus* and *Acidiferrobacter* were found not to belong to any of the three families but were included in the tree as outgroups.

The whole genome-based phylogenetic tree (Figure 1), constructed using unique PGFams as homology groups, supports the ANI analysis and confirms the three groups of strains and species as separate clades in the tree. It also includes representative genomes of chemotrophic species that had previously been considered as belonging to *Ectothio-rhodospiraceae* but are phylogenetically at a distance, which suggests their grouping in a separate new family. The only chemotrophic bacteria that are especially closely related to the phototrophic *Ectothiorhodospiraceae* species, in particular to *Thiorhodospira sibirica*, are species of the two groups of *Thioalkalivibrio* (Figure 1). Of the two distinct groups, one includes, among others, the type species *Thioalkalivibrio versutus* and *Thioalkalivibrio halo-philus*, and the other, among others, *Thioalkalivibrio sulfidophilus* and *Thioalkalivibrio denitrificans* [34].

**Figure 2:**
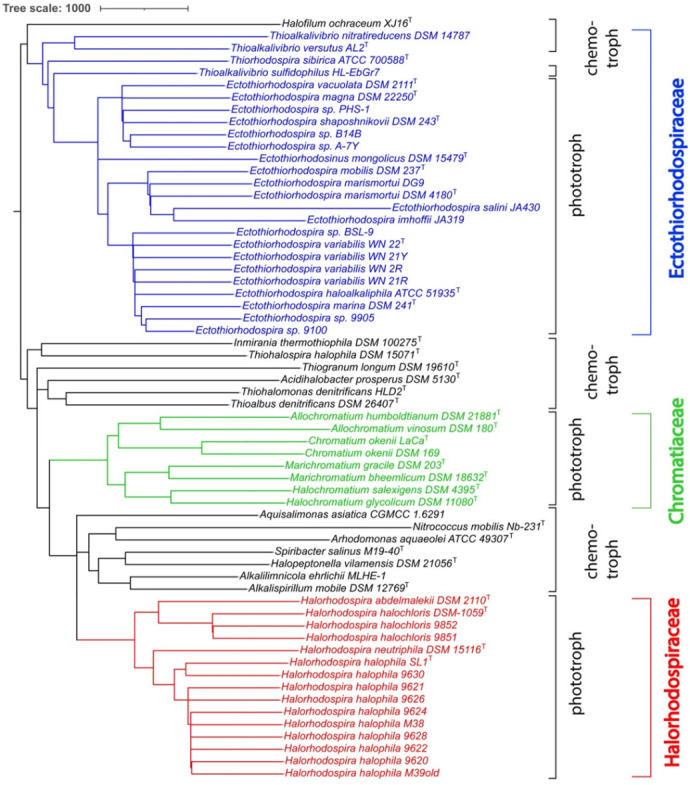
16S rRNA-derived phylogenetic tree for *Ectothiorhodospiraceae* species. The phylogenetic tree was calculated by the neighbour-joining (NJ) method [32] in Jalview [33]. The Jalview NJ method uses the BLOSUM62 substitution matrix to compute a sum of scores for the residue (base) pairs at each aligned position. The length of the branches is proportional to the number of nucleotide substitutions per site. iTOL was used to draw the phylogenetic trees expressed in the Newick phylogenetic tree format [30].

In addition to the whole genome-based tree, we also constructed a 16S rRNA-based phylogenetic tree that includes a comparison of all sequenced phototrophic *Ectothio-rhodospiraceae* (Figure 2), including *Ect. salini* and *Ect. imhoffii*, for which there is no genome sequence available. Included for comparison were 16S rRNAs of the chemotrophic species previously assigned under *Ectothiorhodospiraceae*. The results of the 16S rRNA tree are consistent with the whole genome-based tree and ANI comparisons.

These data show that the type strains of *Ect. variabilis* DSM 21381^T^ (WN 22^T^) and *Ect. haloalkaliphila* ATCC 51935^T^ are highly similar. With an ANI of 98.3%, which is above the suggested 95% species cutoff, they may be regarded belonging to the same species. Both strains originate from soda lakes of the Wadi el-Natrun in Egypt [14,35,36]. Additional isolates from the Wadi el-Natrun (BN9902, WN21Y, WN21R, WN2R) are also highly similar to this species (Table 1 [36]). Despite some earlier results [14,36], 16S rRNA sequences support this high similarity, with 97.2% similarity between the two type strains, but may not necessarily demark species identity. This issue will have to be resolved in more detailed studies.

The results also support the recognition of the following closely related but distinct species. *Ect. marismortui* DSM 4180^T^ is close to *Ect. mobilis* DSM 237^T^ and *Ect. shaposhnikovii* DSM 243^T^ close to *Ect. vacuolata* DSM 2111^T^. Previously, the taxonomic status of *Ect. marismortui* DSM 4180^T^ and *Ect. vacuolata* DSM 2111^T^ were disputed. They were recognized as distinct species by Imhoff [6] and Imhoff and Süling [14], though it was suggested by Ventura et al. [37,38] to combine *Ect. vacuolata* with *Ect. shaposhnikovii* and *Ect. marismortui* with *Ect. mobilis,* primarily based on data on DNA–DNA hybridization and ribotyping. The results of ribotyping, however, did not clearly support this conclusion. They revealed identity between two strains of *Ect. mobilis* (DSM 237^T^ and DSM 240) but clear differences of these to *Ect. marismortui* DSM 4180^T^. They also revealed the identity of two strains of *Ect. vacuolata* (DSM 2111^T^ and B3) with clear differences to *Ect. shaposhnikovii* DSM 243^T^ [37]. In addition, the values of DNA–DNA hybridization were only 80–84% [37]. In contradiction to this suggestion and based on genetic studies of Rubisco and nitrogenase genes, Tourova et al. [39] recognized the four species as distinct species. The presented data on ANI and whole genome-based comparisons are in good agreement with considerations by Imhoff [6] and Tourova et al. [39], recognizing *Ect. vacuolata* DSM 2111^T^ and *Ect. shaposhnikovii* DSM 243^T^ as distinct species with only 90% ANI and also recognizing *Ect. marismortui* DSM 4180^T^ and *Ect. mobilis* DSM 237^T^ as distinct species with ANI values of 93%. Therefore, ANI supports the distinction of these four bacteria at the species level and also confirms the close relationships of the two couples of species.

The results also suggest the assignment of several new isolates to one or the other of the established species. Of two strains that were isolated as arsenite-oxidizing bacteria [40], strain BSL-9 originating from Big Soda Lake represents a distinct new species closely related to *Ect. haloalkaliphila* and *Ect. marina*, while strain PHS-1 originating from Mono Lake is a strain of *Ect. vacuolata* (Figure 1 and Table 2). Strain DG9 is an additional isolate of *Ect. marismortui* from Berikei Sulfur Springs in Dagestan (Russia, [41]). Strains BN9100 (=YC6.1), an isolate from Solar Lake by H. Biebl (Braunschweig, Germany) and BN9905 (=ScotB), isolated from the seashore near Inverary (Scotland) by one of us (JFI), are new isolates of *Ect. marina.* Strains A-7R, A-7Y and B14B form a separate clade on the whole genome phylogenetic tree and have ANI values <90% with respect to any of the other strains, but high ANI values amongst themselves, and therefore represent a new species related to *Ect. shaposhnikovii* and *Ect. vacuolata* (Figure 1 and Table 2).

The present data support the distinction of *Ectothiorhodosinus mongolicus* [42] and also *Thiorhodospira sibirica* [43] in genera separate from *Ectothiorhodospira*. *Ect. magna* [44], though distantly related to other species of the genus, is placed inside a group of species together with *Ect. shaposhnikovii* and *Ect. vacuolata*.

### 3.2. Genomes of Halorhodospira Species

Four *Halorhodospira* species are currently recognized: *Hlr. halophila* [45,46], *Hlr. halochloris* [47], *Hlr. abdelmalekii* [48] and *Hlr. neutriphila* [49]. The genome sequence of *Hlr. halophila* SL1^T^ was previously determined [22] and information on genome sequences of *Hlr. halophila* BN9626, BN9620, BN9630, *Hlr. neutriphila* DSM 15116^T^, *Hlr. halochloris* DSM 1059^T^ and *Hlr. abdelmalekii* DSM 2110^T^ were reported earlier [15,16,17].

We have now added genome sequences of additional strains of *Hlr. halochloris* and *Hlr. halophila*, which originate from the Wadi el-Natrun in Egypt [35,50] and from Mongolian soda lakes (Gorlenko, personal communication) (Table 1). The ANI, whole genome-based and also 16S rRNA-based phylogenetic tree comparison of the *Halorhodospira* species (Table 3, Figure 1 and Figure 2) show a clear distinction of five different species.

The first group consists of *Hlr. halophila* SL1^T^ and *Hlr. halophila* BN9630. The close relationship of strain BN9630 to the *Hlr. halophila* type strain was demonstrated previously by 16S rRNA sequence analyses and fatty acid analyses [9,14] and is now supported by near identity in terms of the ANI (>99%) (Table 3). The 16S rRNA sequences delineated from the genome sequence actually are only two bases different between the two strains.

A second group is formed by strain BN9626 and a number of other isolates (BN9620, BN9621, BN9622, BN9624, BN9628, M38 and M39old), which differed from strain SL1^T^ with respect to fatty acid composition [9] and revealed only 85–86% ANI identity to *Hlr. halophila* SL1^T^ (Table 3). This indicates that these strains could be recognized as strains of a distinct species. The average amino acid identity (AAI) is 89.3% for 2414 orthologues of strains SL1^T^ and BN9626, which is consistent with the ANI. The 16S rRNA identity (obtained from the genome sequences) between *Hlr. halophila* BN9626 and SL1^T^ with 18 differences out of 1537 bp is 98.8%, which is within a range allowing species distinction according to [51] (see systematic conclusions, below). More detailed studies are required to clarify the situation.

Third, according to the whole genome phylogenetic tree (Figure 1) *Hlr. neutriphila* DSM 15166^T^ [49] is clearly distinct from *Hlr. halophila*. The ANI to strains of *Hlr. halophila* is 77.4–77.6%, indicating considerably greater divergence. The 16S rRNA obtained from the genome sequence also averages 54 bases different from that of strains SL1^T^ and BN9626 (96.5% identity), which places *Hlr. neutriphila* on a separate clade on the 16S rRNA tree (Figure 2). The AAI for *Hlr. neutriphila* and strains SL1^T^ and BN9626 is 72.9% (1730 orthologs). The G + C content of the genome sequence from *Hlr. neutriphila* is 72 mol% and thus significantly higher than all other *Halorhodospira* species (Table 1).

Further, according to the genome-based phylogenetic tree, the green-colored, bacteriochlorophyll *b* containing species *Hlr. halochloris* and *Hlr. abdelmalekii* form a major branch distinct from the groups of red-colored species, which have bacteriochlorophyll *a* (Figure 1). The complete genome sequence of *Hlr. halochloris* DSM 1059^T^ (=BN9850^T^) (APO17372) was established by [23] and we have added a second genome sequence of this strain (NRRM00000000). The genome sequence supposedly determined from the *Hlr. halochloris* strain A^T^ (=DSM 1059^T^) by the authors of [52] turned out to be almost identical to the one from *Ect. haloalkaliphila* ATCC 51935^T^ (AJUE00000000) and is not from *Hlr. halochloris*. It could originate from a culture contaminant or a mislabeled culture.

The genomes of the three *Hlr. halochloris* strains (BN9850^T^, BN9851 and BN9852) have a significantly lower G + C content (55.8–56.1 mol%) compared to *Hlr. abdelmalekii* (62.9 mol%) and other *Halorhodospira* species (>67.9 mol%; Table 1). The genome sequence of *Hlr. abdelmalekii* BN9840^T^ (= DSM 2110^T^) is significantly different from *Hlr. halochloris* (ANI of 72.5%), *Hlr. halophila* and *Hlr. neutriphila* (ANI of approximately 73–74%). There are 65 differences (95.8% identity out of 1551 bp) in the 16S rRNA between *Hlr. abdelmalekii* and *Hlr. halochloris,* as obtained from the genome sequences. The 16S rRNA of the green-colored species, *Hlr. halochloris* and *Hlr. abdelmalekii*, on average has 87 differences to the red-colored species. The AAI between the two green-colored species is 71.6% (1856 orthologs) and that for the two green-colored species and the three red-colored species is 68.6%.

### 3.3. Genome-Delineated Properties

#### 3.3.1. Sulfur and Thiosulfate Oxidation

In the *Chromatiaceae,* elemental sulfur is stored in the periplasm inside a proteinaceous membrane made up of one to five sulfur globule proteins rich in alternating Gly and Tyr residues [53,54]. None of the *Ectothiorhodospiraceae* and the *Halorhodospira* species, for that matter, store sulfur intracellularly and contain sulfur globule proteins; the sulfur is transported outside the cells and must be transported back inside for further oxidation, once all the sulfide and thiosulfate are exhausted. It is only then that it is further oxidized to sulfate in the cytoplasm. Detailed studies on processes of sulfur oxidation in purple sulfur bacteria have been made by Dahl and coworkers [55,56].

Most of the *Ectothiorhodospiraceae* species contain the thiosulfate-oxidizing enzymes SoxAXYZB, with the apparent exception of *Ect. magna* and *Trs. sibirica,* that only contain SoxY and Z. The genes are organized in two separate operons. As is apparent from the complete genome of *Ect*. BSL-9 containing a single chromosome, the genes *soxA* and *soxX* are in one location and *soxYZB* in another. In those *Halorhodospira* species that use thiosulfate, the diheme SoxA and SoxX are fused into the single triheme protein, SoxXA. *Hlr. abdelmalekii* and *Hlr. halochloris* do not use thiosulfate as a growth substrate and do not contain Sox enzymes. In *Chromatiaceae*, genes of the thiosulfate-oxidizing enzymes are also located in two operons; however, *soxB* is associated with *soxAX* and the two gene clusters are *soxAXB* and *soxYZ*.

#### 3.3.2. Glutathione

Glutathione is a common small molecule reductant in bacteria, which has several important functional roles [57]. It is present in most bacteria, though in some phototrophic purple bacteria it is replaced by the glutathione amide, in which the terminal glycine carboxylate is amidated, as was first shown for *Marichromatium gracile* [58]. In most bacteria, glutathione reductase has an Arg21 that, according to three-dimensional structural analysis, contributes to the binding of glutathione via a salt bridge to the carboxyl group of the glutathione glycine residue [59]. As found for the glutathione reductase of *Marichromatium gracile* and other *Chromatiaceae*, the Arg21 is substituted by Glu21 and in consequence glutathione, but not its amide, is repelled from the binding site [57]. Thus, glutathione reductase Glu/Arg21 can be used as a proxy for the presence or absence of glutathione amide. We have found through genome sequencing that all *Chromatiaceae* and *Ectothiorhodospiraceae* (with the exceptions of *Ect. mobilis* DSM 237^T^ and both strains of *Ect. marismortui*, DSM 4180^T^ and DG9, where a glutathione reductase has not been found) have Glu21 and are thus likely to have glutathione amide. However, glutathione reductases of all *Halorhodospira* species have Arg21 like the majority of bacteria and are likely to have the normal glutathione.

#### 3.3.3. Carboxysome Genes

The carboxysome is an important structure in many autotrophic bacteria, such as the cyanobacteria [60] and many autotrophic proteobacteria [61]. The carboxysome forms a proteinaceous membrane that encloses Rubisco and carbonic anhydrase and its genes are associated with bicarbonate transporters. The carboxysome shields Rubisco from non-functional reactions with oxygen and provides CO_2_ where it is needed. Though a definite proof for the physical structures in purple sulfur bacteria is lacking, we have found genes of putative carboxysome peptides A and B and carboxysome shell proteins *csoS1*, *csoS2* and *csoS3* in many *Chromatiaceae* and *Ectothiorhodospiraceae*. They are present in seven species of *Ectothiorhodospiraceae* but not in in *Ect. mobilis* DSM 237^T^ and both strains of *Ect. marismortui* (DSM 4180^T^ and DG9)*, Ect.* PHS-1 and *Trs. Sibirica*. Carboxysome genes appear to be absent from *Halorhodospiraceae*, though ribulose bisphosphate carboxylase, phosphoribulose kinase and enzymes involved ini the Calvin Cycle are present.

#### 3.3.4. Nitric Oxide Reduction

As mentioned above, *Ectothiorhodospira* strains A-7Y, A-7R and B14B are closely related but different from *Ect. shaposhnikovii* DSM 243^T^ and *Ect. vacuolata* DSM 2111^T^. These three strains have 15 unique PGFams that are not present in any of the other *Ectothio-rhodospiraceae* genomes and all of them contain a gene cluster for nitric oxide reductase (NOR) that seems to be missing from all the other species. The *norCBQD* cluster contains two NO reductase activation proteins and both the small and large NOR subunits (C and B), which show similarity to cytochrome oxidases. There have been few studies of nitric oxide reduction in the *Ectothiorhodospiraceae*, but these three strains appear to at least have the genetic capability for nitric oxide reduction. In addition to the overall genomic distinction, these genetic differences are another reason to consider these strains as a separate species.

#### 3.3.5. HIPIP

In *Chromatiaceae* the iron–sulfur protein HiPIP was shown to be the electron donor to the photosynthetic reaction center PufLM and the electron acceptor from the BC1 complex [62,63]. The majority of *Ectothiorhodospiraceae* contain either three or four HiPIP isozymes, with the exception of *Ets. mongolicus* and *Trs. sibirica*, which have only one. In addition, *Hlr. halophila* has genes for four HiPIP isoenzymes, two of which are abundant in cell extracts [64]. The gene for one of these isozymes is mixed in with the photosynthetic genes, suggesting that it is the electron donor in this species as it is in *Chromatiaceae* and presumably *Ectothiorhodospiraceae.* It is likely that it acts as a mediator of electrons between the *bc*_1_ complex and photosynthetic reaction center in these bacteria. HiPIP II actually forms a strong complex with the reaction center and rapidly reacts with soluble HiPIP [65]. On the other hand, the green-colored *Halorhodospira* species do not have HiPIP and are likely to utilize cytochrome *c*_5_, which is present in all *Halorhodospira* species characterized to date, but not in the *Ectothiorhodospiraceae*. Cytochrome *c*_5_ is known as a soluble mediator in the green sulfur bacteria in the family *Chlorobiaceae* and the membrane bound *c*_5_ is a possible component of the *bc* complex [66].

In the green-colored *Halorhodospira* species, there are no HiPIP genes at all, which is an important difference to the red-colored *Halorhodospira* species. In this case, the electron mediator between the *bc*_1_ complex and photosynthetic reaction center must be different. Perhaps due to the difficulty in growing *Hlr. halochloris* and *Hlr. abdelmalekii*, no experimental studies of electron transfer have been published. However, all of the *Halorhodospira* species produce a soluble cytochrome *c*_5_ similar to those found in *Chlorobiaceae* species and which is thought to couple the *bc* complex to the photosynthetic reaction center. It is proposed that this cytochrome *c*_5_ is the electron mediator in these two species.

#### 3.3.6. Cytochrome *b*_5_

Cytochrome *b*_5_, which is a well-known constituent of eukaryotic cells, has for the first time found in *Ect. vacuolata*, where its function is unknown [67]. It differs from its eukaryotic counterparts in having a cysteine disulfide and the presence of a signal peptide, which suggests that it is located in the periplasm. At least seven examples are now known from the genome sequences of *Ectothiorhodospiraceae*. These are *Ect. vacuolata* DSM 2111^T^*, Ect.* PHS-1*, Ect. shaposhnikovii* DSM 243^T^*, Ect. mobilis* DSM 237^T^*, Ect.* BSL-9*, Ect. haloalkaliphila* ATCC 51935^T^ and *Ect. marina* DSM 241^T^.

The 10-heme cytochromes, MtrA and MtrF (or OmcA), as well as the outer membrane porin MtrB, are involved in the reduction and solubilization of insoluble iron and manganese minerals primarily in *Shewanella* species, as well as in other bacteria [68]. MtrAB are occasionally found in the purple sulfur bacteria, such as *Ect. vacuolata, Ect. shaposhnikovii, Ect. haloakaliphila, Ect.* BSL-9 and *Ect. marina*. In the species of *Ectothiorhodospiraceae*, these two genes are also associated with OmcA, as in *Shewanella*. Cytochrome *c*_4_ or HiPIP are sometimes found associated with them, suggesting oxidation of FeII rather than reduction of FeIII, since they have high redox potentials. A HiPIP gene is adjacent to the MtrABF cluster in *Ect. vacuolata* DSM 2111^T^ and *Hrs*. *halophila*, although not in other species considered herein.

#### 3.3.7. Arsenic Oxidation

The *Ectothiorhodospiraceae* and the *Halorhodospiraceae* are generally capable of oxidizing arsenic (III) to arsenic (V), but only a few species are capable of using As(III) as sole electron donor for growth, including *Ect*. species strain PHS-1 and *Ect*. species strain BSL-9 [40]. The enzymes involved are the molybdopterin proteins ArxA and ArrA, which are closely related. Most *Ectothiorhodospiraceae* have *arrABC* genes, but only *Ect*. PHS-1, *Ect*. BSL-9, *Ect. shaposhnikovii* DSM 243^T^, *Ect.* B14B, *Ect.* A-7Y, *Ect.* A-7R, and *Hrs. halophila* SL1^T^ and BN9630, have five to eight *arx* genes. The authors of [40] examined four of these species plus *Ect. vacuolata* for oxidation of As(III) and checked for As(III) dependent growth and found that only PHS-1 and BSL-9 would grow with arsenic. Therefore, there has to be more to the story because *Ect. shaposhnikovii* and *Hrs. halophila* have the requisite genes but failed to grow.

#### 3.3.8. Photoactive Yellow Protein—PYP

Another interesting difference between the red- and green-colored *Halorhodospira* strains is that only the red strains have a pair of photoactive yellow proteins (PYP) [69]. It is completely absent in the green-colored strains but has been discovered in the *Thio-rhodospira sibirica* genome as well as *Halochromatium salexigens* (*Chromatiaceae*). The only proven role of PYP in purple bacteria is to reverse the effects of red light on the bacteriophytochrome in the hybrid protein PPR in *Rhodocista centenaria*, which is a PYP/bacteriophytochrome/histidine kinase [70]. The *Chromatiaceae* do not have PPR, but some species do have a hybrid PYP/bacteriophytochrome/diguanylate cyclase/phosphodiesterase, which is called PPD [71]. Interaction among the separate domains has not been demonstrated in PPD and the functional role of the PYP domain is likely to be different than it is in PPR.

#### 3.3.9. Photosynthesis Gene Clusters

**Figure 3:**
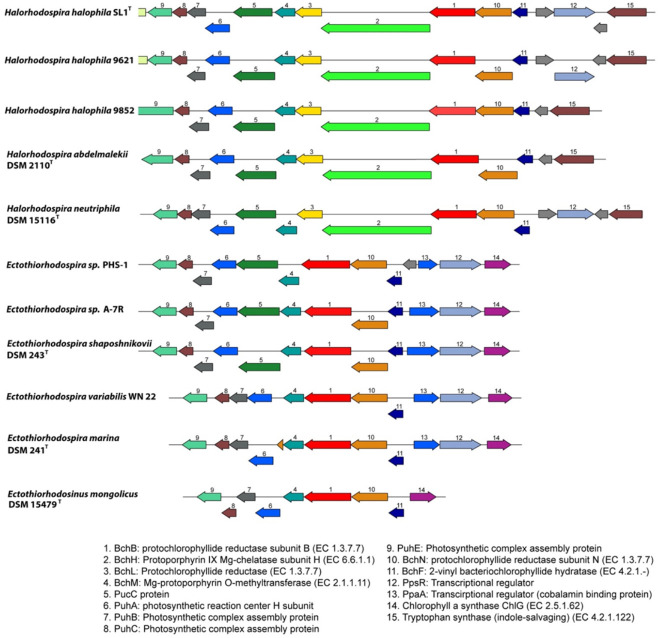
Comparison of the *bch*B genomic region between representatives of *Halorhodospira* and *Ectothiorhodospira* species. Genes are colored based on their family membership.

As other phototrophic *Proteobacteria*, *Ectothiorhodospiraceae* and *Halorhodospira* species have photosynthesis gene clusters, including genes for carotenoid and bacteriochlorophyll biosynthesis, the photosynthetic reaction center and antenna proteins, as well as regulatory and sensory proteins. The structure of the gene clusters and the arrangement of genes show clear differences between *Ectothiorhodospira* and *Halorhodospira* species (Figure 3).

While in *Halorhodospira* species a cluster with *ppsR-bchFNBHLM* genes (the regulator gene *ppsR* is absent from *Hlr. abdelmalekii* and *Hlr. halochloris*) is present, in *Ectothio-rhodospira* species an additional regulatory *ppaA* gene is combined with the *ppsR* gene (genes 12 and 13 in Figure 3).

The presence of the two regulatory genes *ppsR* and *ppaA* in the photosynthetic gene cluster is common to many phototrophic *Alpha*- and *Betaproteobacteria* as well as *Gemmatimonas*. The *ppaA* gene is absent from *Chromatiaceae* and *Halorhodospiraceae* and is found among phototrophic *Gammaproteobacteria* only in the genus *Ectothiorhodospira* (Figure 3). Both regulatory genes are absent from *Ets. Mongolicus.* Quite characteristic for *Ectothio-rhodospiraceae,* and different to most other purple bacteria, including the *Halorhodospiraceae,* is a gene cluster *ChlG-ppsR-ppaA-bchFNB*, with the exclusion of *bchL* and *bchH* genes from the common *bchFNBHLM* gene cluster, as shown for representatives of the genus *Ectothiorhodospira* (Figure 3). Both *bchH* and *bchL* genes are at separate locations in these bacteria. All *Halorhodospira* species have an additional regulator gene *pufQ* which is located between *bchZ* and *pufB* (*bchCXYZ-pufQBALMC*). This regulator is found in many phototrophic *Alphaproteobacteria* but is absent from all *Ectothiorhodospiraceae* and from *Chromatiaceae* as well. All *Halorhodospira* species and *Ectothiorhodospiraceae* lack the aerobic Mg-protoporphyrin IX monomethylester oxidative cyclase (*acsF*) and therefore depend on anoxic conditions for bacteriochlorophyll biosynthesis using the anaerobic form of the enzyme (encoded by *bchlE*).

### 3.4. Habitats and Environmental Distribution

Quite remarkably, species of phototrophic *Ectothiorhodospiraceae* and *Halorhodospiraceae* (*Ectothiorhodospira, Halorhodospira*, *Thiorhodospira* and *Ectothiorhodosinus* species), including their chemotrophic relatives, are characteristic inhabitants of marine and saline waters worldwide and preferably develop in alkaline and saline soda lakes. They are phylogenetically related to alkaliphilic chemotrophic sulfur-oxidizing bacteria of the genera *Thioalkalivibrio*, *Alkalilimnicola*, *Alkalispirillum* and are important sulfur-oxidizing chemotrophic bacteria in many alkaline soda lakes [72,73]. A recent review summarizes their occurrence in various types of salt and soda lakes in different geographic regions [74].

Although *Halorhodospira halophila* was first isolated from Summer Lake, Oregon [45,46] and *Hlr. neutriphila* originates from a marine saltern [49], the great majority of the studied strains originate from African soda lakes, most prominently those of the Wadi el-Natrun. This contains all strains of the green-colored species and a second strain of *Hlr. halophila* (9630). Several other strains assigned to this species (including, among others, BN9620, BN9622 and BN9624) are likely distinct on the species level from *Hlr. halophila*. In addition to strains from the Wadi el-Natrun, two isolates from Mongolian soda lakes (M38 and M39old) belong to this presumably new species.

The first intensively studied soda lakes with mass developments of red-colored and green-colored *Halorhodospira* species were those in the Wadi el-Natrun in Egypt [35,50,75]. While pH-optima of the type strain of *Hlr. halophila* SL1^T^ (isolated from the highly alkaline and saline Summer Lake in Oregon) were found to be at 7.4–7.9 [45,46], isolates from the Wadi el-Natrun had pH optima at 8.5–9.0. Two more haloalkaliphilic species, the green-colored, bacteriochlorophyll *b*-producing *Hlr. halochloris* [47] and *Hlr. abdelmalekii* [48], originate from these soda lakes.

In addition, isolates of the moderate halophilic and alkaliphilic *Ectothiorhodospira* species *Ect. haloalkaliphila* [14,35] and *Ect. variabilis,* which is most closely related to *Ect. halo-alkaliphila* [36], were found in soda lakes of the Wadi el-Natrun and also in soda lakes from Siberia and Mongolia [36].

*Halorhodospira halophila* and two morphological distinct bacteria assigned to the genus *Ectothiorhodospira* were present in Mongolian soda lakes with high salinities (>15% salts), while other species of *Ectothiorhodospira* and *Thiorhodospira* were isolated from lakes with lower salinities of up to 5.5–6.0% salts. For the first time the moderately halophilic *Ectothiorhodosinus mongolicus* (salt optimum 1–7%) was isolated from one of these lakes [42]. Additionally, *Thiorhodospira sibirica* [43] and *Ect. magna* [44] were isolated from soda lakes of remote areas in Asia in Siberia, Mongolia and the Transbaikal region, but have so far not been found in other locations. Possibly the remote origin of these bacteria indicates their separate evolution and is a reason for their distant relationship to other strains of the *Ectothio-rhodospiraceae/Halorhodospiraceae* families.

Other *Ectothiorhodospira* species, especially *Ect. marina* and *Ect. mobilis*, prefer marine habitats, where they have been regularly observed and repeatedly been isolated from.

In recent years, proof of the presence of *Ectothiorhodospiraceae* in alkaline and saline lakes worldwide has been obtained by analysis of clone libraries. The analysis of 16S rRNA gene clone libraries from several lakes of the Wadi el-Natrun (Lake Fazda, Lake Hamra and Lake UmRisha) revealed a great diversity of sequences related to *Ectothio-rhodospiraceae* species, including *Hlr. halochloris*, and *Ect. haloalkaliphila* [76]. In addition, communities of phototrophic purple bacteria in Chilean salt lakes of the Atacama Desert, Laguna Chaxa and Laguna Tebenquiche that were studied by clone libraries of *pufLM* gene sequences were found to contain bacteria related to *Ect. mobilis, Ect. variabilis* and *Hlr. halophila* as closest relatives [77,78].

## 4. Systematic Conclusions

The comparison of a large number of genome sequences of phototrophic purple sulfur bacteria in the present study clearly demonstrated the need to separate *Halorhodospira* species and relatives from the *Ectothiorhodospiraceae* and to place them in the new family *Halorhodospiraceae*. The comparison of the genomic phylogeny, including considerations of ANI, demonstrated that the phototrophic *Ectothiorhodospiraceae* as currently known represent two separate families, being almost equally distant from the *Chromatiaceae* family. Based on the significant differences of the extremely halophilic species from all other *Ecto-thiorhodospiraceae*, we propose to recognize these species as members of a new family, the *Halorhodospiraceae* fam. nov.

The herein proposed reclassification of the families of phototrophic purple sulfur bacteria with the separation of *Halorhodospira* species from the *Ectothiorhodospiraceae* family and the existence of three families of purple sulfur bacteria within the *Gammaproteobacteria* requires a careful reconsideration of species assignment to these families and sheds new light on the assignment of a number of chemotrophic species and genera to the *Ectothiorhodospiraceae* which have been made in recent years but do not warrant to be included within this family.

Among those bacteria that have been assigned to the *Ectothiorhodospiraceae* which without doubt cannot be considered as members of this family are *Acidiferrobacter thio-oxydans* [79], now classified with *Acidiferribacteraceae* and *Acidiferribacterales* [80,81] and *Oceanococcus atlanticus* [82]. They do not fit into any of the three phototrophic *Chromatiales* families (Figure 1). In addition, *Halofilum ochraceum* [83], with ANI values to all considered *Ectothiorhodospiraceae* of only 65–68%, should not be included in *Ectothiorhodospiraceae* or *Halorhodospiraceae*. In addition, a few bacteria for which genome sequences at present are not available, should not be included in *Ectothiorhodospiraceae* or *Halorhodospiraceae* based on information available for 16S rRNA gene sequences and other properties. One of these species is *Natronocella acetinitrilica* [84]. Two other species are *Methylonatrum kenyense*, which is a gammaproteobacterium considered to be of unknown affiliation (genus incertae sedis), and *Methylohalomonas lacus* [85], which may be related to *Thioalkali-spira* species of the *Thioalkalispiraceae* [86].

Other species/genera that have been assigned to the *Ectothiorhodospiraceae* are distantly related to the phototrophic *Chromatiaceae, Ectothiorhodospiraceae* or *Halorhodospiraceae* by deep-branching lineages both in genome-based and 16S rRNA-based phylogenetic trees (Figure 1 and Figure 2). These are *Thiohalospira halophila*, *Thiohalomonas denitrificans*, *Thiogranum longum*, *Thioalbus denitrificans*, *Inmirania thermothiophila* and the acidiphilic *Acidihalobacter prosperous* and related species, which therefore should not be considered as belonging to either *Ectothiorhodospiraceae* or *Halorhodospiraceae*. Their placement in one or more new separate families needs to be evaluated and these bacteria will not be further considered in the present discussion.

Halophilic and alkaliphilic chemotrophic bacteria that are most closely related to phototrophic *Ectothiorhodospiraceae* are species of the genus *Thioalkalivibrio*, particularly those belonging to the *Thioalkalivibrio sulfidophilus* cluster (Figure 1 and Figure 2). Species of both clusters of *Thioalkalivibrio* are the only known chemotrophic bacteria that can be con-sidered to be included in the *Ectothiorhodospiraceae*.

The new family *Halorhodospiraceae* is represented by two major groups of phototrophic bacteria, the red-colored *Hlr. halophila* and relatives and the green-colored *Hlr. halochloris* and relatives. Phenotypic and phylogenetic differences suggest a separation of the green-colored species in a separate genus, with ANI values of the type species of 70.4–71.3 to strains of *Hlr. halophila*. Although *Hlr. neutriphila* appears phylogenetically and phenotypically (characteristically by the preference for neutral pH and high G + C content of 72 mol%) distinct from *Hlr. halophila*, an ANI >77% to *Hlr. halophila* (Table 3) and 16S rRNA identity of 96.5% to the type strain of *Hlr. halophila* SL1 support recognizing this bacterium as a distinct species of the genus *Halorhodospira*. This is in line with proposals made by others suggesting genus delineations at ANI values close to 74% [51,87].

According to both the phylogenomic and the 16S rRNA tree, a phylogenetic cluster distinct to these phototrophic bacteria is represented by a group of chemotrophic bacteria, including chemotrophic alkaliphilic and halotolerant species, the nitrifying *Nitrococcus mobilis*, *Aquisalimonas asiatica*, *Spiribacter salinus*, *Spiribacter (Halopeptonella) vilamensis* and *Arhodomonas aquaeolei* (Figure 1 and Figure 2). According to 16S rRNA phylogeny, this cluster includes *Alkalispirillum mobilis* and *Alkalilimnicola ehrlichii* and relatives as well, which, according to the genomic tree, form a separate lineage closer to *Halorhodospira*. The phylogenetic distance of the whole group of chemotrophic relatives suggests the exclusion of these bacteria from the *Halorhodospiraceae* and placing them within one or more separate new families. Accordingly, the *Halorhodospiraceae* are represented exclusively by phototrophic bacteria.

Based on the present data, *Ectothiorhodosinus mongolicus* [42] and *Thiorhodospira sibirica* [43] represent genera distinct from *Ectothiorhodospira*. *Ect. magna* [44], though distantly related to other species of the genus, is placed inside a group of species together with *Ect. shaposhnikovii* and *Ect. vacuolata*. ANI values among all studied strains/species of the genus *Ectothiorhodospira* are >74.9% which is in line with a proposed genus demarcation of approximately 74% [88]. Other strains (strains A-7Y, A-7R and B14B) with ANI values to related *Ectothiorhodospira* species below 90% (87.8–89.4) presumably represent a new species of this genus. Although little information is known, and genome sequences are not available for *Ect. salini* and “*Ect. imhoffii*”, both species appear most closely related to *Ect. mobilis* and *Ect. marismotui* according to 16S rRNA gene sequences (Figure 2; [88,89]).

Characteristics of the three families.

There are three families of the *Chromatiales* that are characterized by the presence of phototrophic purple sulfur bacteria forming coherent clusters that are, with very few exceptions, clearly distinct from their chemotrophic relatives. These families can be distinguished by a number of phenotypic properties as well as on the basis of genomic properties.

The *Chromatiaceae* primarily live in fresh water or marine habitats; they are phototrophic and primarily autotrophic. When oxidizing sulfide and thiosulfate, intermediate sulfur is stored in the periplasmic space, enclosed within a proteinaceous membrane containing one to five small, related sulfur globule proteins. The thiosulfate dehydrogenase, SoxA, and its electron-acceptor, SoxX, exist as separate subunits. The small protective thiol, glutathione, is present as the amide.

*Ectothiorhodospiraceae* primarily live in shallow alkaline desert soda lakes and also marine shallow waters and tolerate up to about 5–7% salt. They are primarily phototrophic and autotrophic. When oxidizing sulfide and thiosulfate, the intermediate sulfur is excreted into the growth medium without a membrane. SoxA and SoxX exist as separate subunits. Glutathione is present as the amide.

The *Halorhodospiraceae* are found in soda lakes, require elevated salt concentrations for growth and some species can even live in saturated brines. Intermediate elemental sulfur is excreted into the growth medium. SoxX is fused to the N-terminus of SoxA. Glutathione is not amidated. They are phototrophic bacteria forming two distinct groups of species, red-colored species producing bacteriochlorophyll *a* and green-colored species producing bacteriochlorophyll *b*. The red-colored species likely use the small iron–sulfur protein HiPIP as mediator between the *bc*_1_ complex and the photosynthetic reaction center PufLM. On the other hand, the green species are likely to use the small soluble cytochrome *c*_5_ as mediator.

## 5. Emended Description of the Family *Ectothiorhodospiraceae* Imhoff 1984a, 33^VP^

Ec.to.thi’o.rho.do.spi.ra’ce.ae. M.L. fem. n. *Ectothiorhodospira* type genus of the family; -aceae ending to denote a family; M. L. fem. pl. n. Ectothiorhodospiraceae the *Ectothio-rhodospira* family.

The family constitutes slightly or moderately halophilic phototrophic bacteria growing under alkaline conditions and their chemotrophic relatives. They are Gram-negative and belong to the *Gammaproteobacteria*. Cells are spiral-, vibrioid- or rod-shaped, motile by means of polar flagella and divide by binary fission. They are either phototrophic purple sulfur bacteria that perform anoxygenic photosynthesis with bacteriochlorophylls and carotenoids as photosynthetic pigments or aerobic chemolithotrophic sulfur-oxidizing bacteria, some of which may use nitrate as alternative electron acceptors. Growth of phototrophic representatives is preferably anaerobic in the light, with reduced sulfur compounds as electron donors. Sulfide is oxidized to elemental sulfur and is deposited outside the cells, eventually also in the peripheral periplasmic space of the cell body. The final oxidation product is sulfate. *Ectothiorhodospiraceae* are found in marine and moderately saline environments containing sulfide and having an alkaline to extremely alkaline pH. Glycine betaine, but not ectoine, is the major compatible solute in these bacteria.

The mol% G + C of the DNA is from 53.8–68.4 (genome sequence).

Type genus: *Ectothiorhodospira* Pelsh 1936, 120.

## 6. Description of the Family *Halorhodospiraceae*. fam. nov.

Ha.lo.rho.do.spi.ra’ce.ae. M.L. fem. n. *Halorhodospira* type genus of the family; -aceae ending to denote a family; M. L. fem. pl. n. *Halorhodospiraceae* the family of *Halorhodospira.*

The family constitutes moderately or extremely halophilic and extremely halotolerant phototrophic bacteria growing under alkaline conditions. They are Gram-negative and belong to the *Gammaproteobacteria*. Cells are spiral-, vibrioid- or rod-shaped, motile by means of polar flagella and divide by binary fission. They are phototrophic purple sulfur bacteria that perform anoxygenic photosynthesis with bacteriochlorophylls and carotenoids as photosynthetic pigments and have internal photosynthetic membranes as lamellar stacks continuous with the cytoplasmic membrane. Photosynthetic pigments are bacteriochlorophyll *a* or *b* and carotenoids. Growth of phototrophic representatives is preferably anaerobic in the light, with reduced sulfur compounds as electron donors. Sulfide is oxidized to elemental sulfur and is deposited outside the cells. The final oxidation product is sulfate. *Halorhodospiraceae* are found in saline, preferably extremely saline environments containing sulfide and having an alkaline to extremely alkaline pH. They are regular inhabitants and represent major groups of the bacterial populations of soda lakes of various salinities. Species of this family are the most halophilic eubacteria. Glycine betaine, ectoine and trehalose accumulate as compatible solutes in response to salt and osmotic stress.

The mol% G + C of the DNA is 55.8–72.0 (genome sequence).

Type genus: *Halorhodospira* Imhoff and Süling 1996, 112; Imhoff and Süling 1997, 915.VP.

### 6.1. Emended Description of the Genus Halorhodospira

Ha’lo.rho’do. spi’ra. Gr.gen. n. *halos* of the salt; Gr. n. *rhodon* the rose; Gr. n. *spira* the spiral; M.L fem. n. *Halorhodospira*, the spiral rose from salt lakes.

Cells are spiral- or rod-shaped, 0.6–1.2 µm in diameter, motile by bipolar flagella and multiply by binary fission. They are Gram-negative and belong to the *Gammaproteobacteria* and grow photoautotrophically under anoxic conditions with reduced sulfur compounds as electron donors, or photoheterotrophically with a limited number of simple organic compounds. Sulfide is oxidized to elemental sulfur, which is deposited outside the cells and may be further oxidized to sulfate. Internal photosynthetic membranes appear as lamellar stacks continuous with the cytoplasmic membrane. Photosynthetic pigments are bacteriochlorophyll *a* and carotenoids. Growth is dependent on highly saline and alkaline conditions. Greater than 10% (*w*/*v*) total salt concentration is required for optimal growth in all known species, some of which even grow in saturated salt solutions. Glycine betaine, ectoine and trehalose accumulate as compatible solutes in response to salt and osmotic stress. Growth factors are not required. Storage products are polysaccharides, poly-β-hydroxybutyrate and polyphosphate. *Halorhodospira* species are found in hypersaline and extremely saline environments, preferably with moderately to extremely alkaline pH (up to pH 11–12), that contain sulfide and are exposed to light, such as salt flats, salt lakes and soda lakes, but some species may also inhabit salterns and coastal lagoons.

The mol% G + C of the DNA is from 67.9–72.0 (genome sequence).

Type species: *Halorhodospira (Hlr.) halophila* (Raymond and Sistrom) Imhoff and Süling 1996, 110.

### 6.2. Description of the Genus Halochlorospira gen. nov.

Ha’lo.chlo’ro. spi’ra. Gr.gen. n. *halos* of the salt; Gr. n. *chloros* green; Gr. n. *spira* the spiral; M.L fem. n. *Halochlorospira*, the green spiral from salt lakes.

Cells are spiral- or rod-shaped, 0.5–1.2 µm in diameter, motile by bipolar flagella and multiply by binary fission. They are Gram-negative and belong to the *Gammaproteobacteria* and grow photoautotrophically under anoxic conditions with reduced sulfur compounds as electron donors, or photoheterotrophically with a limited number of simple organic compounds. Internal photosynthetic membranes appear as lamellar stacks continuous with the cytoplasmic membrane. Photosynthetic pigments are bacteriochlorophyll *b* and carotenoids. Sulfide is oxidized to elemental sulfur, which is deposited outside the cells and may be further oxidized to sulfate. Growth is dependent on highly saline and alkaline conditions. Greater than 10% (*w*/*v*) total salt concentration is required for optimal growth by all known species, some of which grow in saturated salt solutions. Glycine betaine, ectoine and trehalose accumulate as compatible solutes in response to salt and osmotic stress. Growth factors not required. Storage products are polysaccharides, poly-β-hydroxybutyrate and polyphosphate. *Halochlorospira* species are found in hypersaline and extremely saline environments with slightly to extremely alkaline pH (up to pH 11–12) that contain sulfide and are exposed to light, such as salt flats, salt lakes and soda lakes.

The mol% G + C of the DNA is from 55.8–62.9 (genome sequence).

Type species: *Halochlorospira (Hcs.) halochloris* (Imhoff and Trüper, 1977).

### 6.3. Description of Halochlorospira halochloris comb. nov.

The description is entirely the same as for *Halorhodospira halochloris*.

### 6.4. Descrition of Halochlorospira abdelmalekii comb. nov.

The description is entirely the same as for *Halorhodospira abdelmalekii*.

## Figures and Tables

**Table 1:** Comparison of genome features of the *Ectothiorhodospiraceae* genomes in this study. The genomes are colored by groups as described in the text.

Genome Name	Strain	Alternate Strain	Type Strain	GenBank Accessions	Contigs	Genome Length	GC mol%	quinone	CDS	Coarse Consis-tency	Fine Consis-tency	Sequencing Depth	Reference
*Ectothiorhodospira haloalkaliphila*	ATCC 51935		yes	AJUE00000000	36	3,445,226	63.0	MK-7/Q-8	3155	95.3	93.9	Unknown	[20]
*Ectothiorhodospira haloalkaliphila*	9902	strain C	no	JAJNQR000000000	243	3,666,279	62.7		3602	95.4	93.7	106x	*this study*
*Ectothiorhodospira variabilis*	DSM 21381	WN 22	yes	JAJNAO000000000	84	3,416,336	63.0		3250	94.3	92.8	119x	*this study*
*Ectothiorhodospira variabilis*		WN 21Y	no	JAJNAP000000000	84	3,452,695	63.0		3302	94.3	92.9	145x	*this study*
*Ectothiorhodospira variabilis*		WN 21R	no	JAJNAQ000000000	71	3,484,384	63.0		3341	94.3	92.9	119x	*this study*
*Ectothiorhodospira variabilis*		WN 2R	no	JAJNAR000000000	73	3,484,893	63.0		3341	94.3	92.9	132x	*this study*
*Ectothiorhodospira sp.*	BSL-9		no	CP011994	1	3,550,080	63.0		3350	95.7	93.1	298x	[21]
*Ectothiorhodospira sp.*	9100	YC6.1	no	JAJNAN000000000	161	3,238,183	62.3		3169	93.3	92	103x	*this study*
*Ectothiorhodospira sp.*	9905		no	JAJNAM000000000	180	3,202,442	62.3		3120	94.8	93.4	52x	*this study*
*Ectothiorhodospira marina*	DSM 241	9914	yes	FOAA00000000	41	3,185,852	62.3	MK-7/Q-8	3059	94.8	93.9	378x	[17]
*Ectothiorhodospira mobilis*	DSM 237		yes	NRSK01000000	119	2,796,053	68.3	MK-7/Q-8	2714	95.4	93.1	539x	[17]
*Ectothiorhodospira marismortui*	DSM 4180	9410	yes	FOUO00000000	35	2,624,954	68.2	MK-7/Q-8	2474	94.6	93.7	363x	[17]
*Ectothiorhodospira marismortui*	DG9		no	JAJOZD000000000	40	2,682,160	68.4		2522	94.7	93.7	139x	*this study*
*Ectothiorhodospira vacuolata*	DSM 2111	Beta 1	yes	JAJMLZ000000000	40	3,341,893	63.4	MK-7/Q-7	3100	93.7	92.5	125x	*this study*
*Ectothiorhodospira sp.*	PHS-1		no	AGBG00000000	114	2,943,210	63.7		2725	95.6	94.2	42x	[19]
*Ectothiorhodospira shaposhnikovii*	DSM 243	9710	yes	NRSM01000000	154	3,788,226	62.4	MK-7/Q-7	3743	96	93.9	160x	[17]
*Ectothiorhodospira sp.*	A-7R		no	JAJNQO000000000	47	3,282,361	63.0		3009	96.1	94.4	84x	*this study*
*Ectothiorhodospira sp.*	A-7Y		no	JAJNQN000000000	50	3,282,916	63.0		3019	95.9	94.3	52x	*this study*
*Ectothiorhodospira sp.*	B14B		no	JAJNQM000000000	164	3,422,169	62.9		3209	95.5	93	10x	*this study*
*Ectothiorhodospira magna*	DSM 22250	B7-7	yes	FOFO00000000	58	2,721,342	60.9		2559	94.7	93.6	438x	[17]
*Ectothiorhodosinus mongolicus*	DSM15479	M9	yes	FTPK00000000	6	1,990,961	55.6		1867	95.2	94.5	593x	[17]
*Thiorhodospira sibirica*	ATCC 700588		yes	AGFD00000000	186	3,188,869	56.7		2842	93	92.1	30x	[18]
*Halorhodospira abdelmalekii*	DSM 2110		yes	NRRN01000000	158	3,090,835	62.9	MK-4	2914	94.4	93.2	122x	[17]
*Halorhodospira halochloris*	DSM 1059	9850	yes	AP017372	1	2,819,782	55.8	MK-4	2699	94.4	93.7	123x	[23]
*Halorhodospira halochloris*		9851	no	JAJNRA000000000	141	2,883,546	56.1	MK-4	2847	94.1	93.3	136x	*this study*
*Halorhodospira halochloris*		9852	no	JAJNRB000000000	188	2,888,425	56.1		2861	93.6	92.8	91x	*this study*
*Halorhodospira neutriphila*	DSM 15116		yes	NRSH01000000	287	2,394,163	72.0		2535	95.5	92.3	46x	[17]
*Halorhodospira halophila*	DSM 244	SL1	yes	CP000544	1	2,678,452	68.0	MK-8	2414	99.7	99.3	Unknown	[22]
*Halorhodospira halophila*		9630	no	NRRO00000000	45	2,682,427	67.9	MK-8	2522	99.6	98.9	220x	[17]
*Halorhodospira halophila*		9620	no	NHSE01000000	99	2,703,685	68.4		2600	99.2	98.3	49x	[17]
*Halorhodospira halophila*		9621	no	JAJNRD000000000	87	2,912,850	67.9		2705	99.6	99	70x	*this study*
*Halorhodospira halophila*		9622	no	JAJNRE000000000	121	2,909,722	67.9		2776	99.6	99	80x	*this study*
*Halorhodospira halophila*		9624	no	JAJNRC000000000	20	2,779,891	68.1	MK-8	2599	97.6	96.6	117x	*this study*
*Halorhodospira halophila*		9626	no	NHSN01000000	45	2,857,375	68.2		2720	99.5	98.9	72x	[17]
*Halorhodospira halophila*		9628	no	JAJNRF000000000	27	2,787,144	68.1		2598	99.4	99	68x	*this study*
*Halorhodospira halophila*	M38		no	JAJNQP000000000	101	2,748,046	68.3		2572	99.4	98.8	143x	*this study*
*Halorhodospira halophila*	M39old		no	JAJNQQ000000000	105	2,747,719	68.3		2571	99.4	98.8	135x	*this study*

**Table 2:** Average percentage nucleotide identity (ANI) between pairs of genomes. ANI values >80% are colored and values above the species cutoff (>95%) are presented in bold.

*Ect. vacuolata* DSM 2111																			
**96.3**	*Ect.* PHS-1																		
90.5	90.3	*Ect. shaposhnikovii* DSM 243																	
89.4	89.1	88.1	*Ect.* B14B																
89.2	88.7	87.8	**99**	*Ect.* A-7Y															
88.9	88.7	87.8	**99.1**	**99.9**	*Ect.* A-7R														
86.2	85.9	84.6	83.4	83.3	83.6	*Ect. magna* DSM 22250													
76.8	76.8	76.5	76.4	76.4	76.5	76	*Ect.* BSL-9												
76.7	76.6	76.4	76	76.1	76.3	75.9	91.5	*Ect. haloalkaliphila* ATCC 51935											
76.5	76.5	76.2	76	76.1	76.1	76	91.3	**98**	*Ect. haloalkaliphila* 9902										
76.6	76.6	76.6	75.9	76	76.2	75.8	91.2	**98.3**	**98.2**	*Ect. variabilis* WN 22									
76.1	76.1	76	75.7	75.9	76.1	75.5	86.9	86.8	86.8	86.6	*Ect. marina* DSM 241								
76	76.3	75.6	75.5	75.7	75.9	75.5	87.3	86.9	86.8	86.7	**96.7**	*Ect.* YC6.1							
75.9	76.1	75.7	75.7	75.7	75.8	75.5	87.2	86.8	86.8	86.7	**96.8**	**99.9**	*Ect.* 9905						
76	75.8	75.4	75.1	75	75.3	74.9	76.7	76.2	76.3	75.9	76.1	75.9	75.9	*Ect. mobilis* DSM 237					
75.9	75.8	75.4	75	75	75.3	74.9	76.7	76.4	76.4	76.1	76.1	76	76	92.6	*Ect. marismortui* DSM 4180				
75.5	75.7	75	75	75	75.3	74.9	76.2	75.9	76.2	75.9	76	76	76	92.8	**98**	*Ect. marismortui* DG9			
70	70	69.8	69.9	69.8	69.9	69.9	70.2	70.2	70.1	70.1	70.1	70	70	69.5	69.3	69.5	*Ets. mongolicus* DSM 15479		
69.9	69.8	69.8	69.7	69.5	69.7	70	69.5	69.3	69.3	69.3	69.2	69.5	69.4	69.5	69.3	69.2	68.3	*Trs. siberica* ATCC700588	

**Table 3:** ANI, average percentage nucleotide identity between pairs of genomes. ANI values >90% are colored and values above the species cutoff (>95%) are presented in bold.

*Hlr. halophila* SL1															
**99.99**	*Hlr. halophila* BN9630														
85.8	85.8	*Hlr. halophila* BN9626													
85.8	85.8	**98**	*Hlr. halophila* BN9620												
85.7	85.7	**98.5**	**98.1**	*Hlr. halophila* BN9628											
85.7	85.7	**98.5**	**98.1**	**100**	*Hlr. halophila* BN9624										
85.7	85.7	**98.1**	**98.5**	**98.4**	**98.4**	*Hlr. halophila* BN9622									
85.7	85.7	**98.2**	**97.9**	**98.7**	**98.7**	**98.1**	*Hlr. halophila* BN9621								
85.6	85.5	**97.4**	**97.9**	**97.4**	**97.4**	**98**	**97.3**	*Hlr. halophila* M39old							
85.6	85.5	**97.3**	**97.8**	**97.4**	**97.4**	**98**	**97.3**	**100**	*Hlr. halophila* M38						
77.4	77.4	77.4	77.4	77.6	77.6	77.6	77.6	77.7	77.6	*Hlr. neutriphila* DSM 15116					
73.3	73.3	73.7	73.7	73.6	73.6	73.6	73.5	73.4	73.4	74.2	*Hlr. abdelmalekii* BN9840				
70.6	70.5	70.9	71.1	71.2	71.2	71.3	71	70.8	70.8	70.7	72.7	*Hlr. halochloris* BN9852			
70.6	70.5	70.7	70.9	71	71	71	70.2	70.7	70.8	70.7	72.5	**97.6**	*Hlr. halochloris* BN9850		
70.5	70.4	70.7	71	70.9	70.9	70.8	71	70.7	70.8	70.6	72.5	**97.6**	**99.94**	*Hlr halochloris* BN9851	

## Data Availability

The Whole Genome Shotgun projects have been deposited at DDBJ/ENA/GenBank under the accession numbers provided in Table 1.
